# Basic Hallmarks of Urothelial Cancer Unleashed in Primary Uroepithelium by Interference with the Epigenetic Master Regulator ***ODC1***

**DOI:** 10.1038/s41598-020-60796-8

**Published:** 2020-03-02

**Authors:** Lars Erichsen, Hans-Helge Seifert, Wolfgang A. Schulz, Michèle J. Hoffmann, Günter Niegisch, Marcos J. Araúzo-Bravo, Marcelo L. Bendhack, Cedric Poyet, Thomas Hermanns, Agnes Beermann, Mohamed Hassan, Lisa Theis, Wardah Mahmood, Simeon Santourlidis

**Affiliations:** 1 Epigenetics Core Laboratory, Institute of Transplantation Diagnostics and Cell Therapeutics, Medical Faculty, Heinrich-Heine University Duesseldorf, Moorenstr. 5, 40225 Duesseldorf, Germany; 2 Department of Urology, University Hospital Basel, Basel, Switzerland; 3 Department of Urology, Medical Faculty, Heinrich-Heine University Duesseldorf, Duesseldorf, Germany; 4 Group of Computational Biology and Systems Biomedicine, Biodonostia Health Research Institute, 20014, San Sebastián, Spain; 5 IKERBASQUE, Basque Foundation for Science, 48009 Bilbao, Spain; 6 Department of Urology, University Hospital, Positivo University, Curitiba, Brazil; 7 Department of Urology, University Hospital, University of Zurich, Zurich, Switzerland; 8 Department of Surgery, Tulane University School of Medicine, New Orleans, LA 70112 USA; 9 Institut National de la Santé et de la Recherché Médicale, University of Strasbourg, 67000 Strasbourg, France

**Keywords:** Bladder cancer, Bladder

## Abstract

Urothelial carcinoma (UC) is a common disease causing significant morbidity and mortality as well as considerable costs for health systems. Extensive aberrant methylation of DNA is broadly documented in early UC, contributing to genetic instability, altered gene expression and tumor progression. However the triggers initiating aberrant methylation are unknown. Recently we discovered that several genes encoding key enzymes of methyl group and polyamine metabolism, including Ornithine Decarboxylase 1 *(ODC1)*, are affected by DNA methylation in early stage UC. In this study, we investigated the hypothesis that these epigenetic alterations act in a feed-forward fashion to promote aberrant DNA methylation in UC. We demonstrate that siRNA-mediated knockdown of *ODC1* expression elicits genome-wide LINE-1 demethylation, induction of LINE-1 transcripts and double-strand DNA breaks and decreases viability in primary cultured uroepithelial cells. Similarly, following siRNA-mediated knockdown of *ODC1*, UC cells undergo double-strand DNA breaks and apoptosis. Collectively, our findings provide evidence that *ODC1* gene hypermethylation could be a starting point for the onset of genome-wide epigenetic aberrations in urothelial carcinogenesis. Furthermore, LINE-1 induction enabled by *ODC1* interference provides a new experimental model to study mechanisms and consequences of LINE-1 activation in the etiology and progression of UC as well as presumably other cancers.

## Introduction

Urothelial carcinoma (UC), the most common cancer of the urinary bladder, is a frequent disease with yearly 549,393 new cases and 199,922 deaths worldwide^1^. Relapse rates of 30–70% and rates of progression at 10–30% for high-grade tumors present a serious health care challenge and impose enormous financial burdens on the health care systems^2^. In particular, UC that have invaded the muscle-layers of the bladder are highly lethal.

Tobacco smoking is thought to contribute to 50% of all UC cases as a risk factor^3^. Aromatic amines such as 2-naphthylamin and polycyclic aromatic hydrocarbons in cigarette smoke cause mutations in key cancer-related genes by forming DNA adducts^4^. Accordingly, exposure to tobacco smoke alone is sufficient to induce transformation in urinary tract epithelial cells, which is moreover accompanied by aberrant DNA methylation^5^. Therefore urologists are advised to inform patients on the factors causing UC and strongly counsel them to stop smoking^6^.

The methylome which consists of all methylated cytosine-guanosine (CpG) dinucleotides, forms an elaborate, plastic and cell-type specific pattern in mammalian cells. It supports genome organization, gene regulation and preserves genome integrity by repressing transposable genetic elements^7^. Importantly, the methylome is influenced by exogenous factors.

Aberrant changes of the genome-wide DNA methylation pattern are broadly-documented and have long been established as an early event in the development of UC^8–10^. Focal DNA methylation changes affect among others tumor suppressor genes^11^ and may therefore causally contribute to the development and progression of UC. Global DNA hypomethylation, a genome-wide decrease in methylcytosine, occurs concomitantly, affecting in particular endogenous retroviruses (HERVs) and retrotransposons (SINEs and LINEs) and may result in their transcriptional reactivation^12^. In particular, substantial global LINE-1 DNA hypomethylation in bladder cancer is accompanied by a shift toward expression of full-length LINE-1 elements^13^.

Expression of retrotransposons can damage the genome^14^. LINE-1 RNA and protein overexpression, e.g., have been linked to apoptosis, DNA damage and repair, tumor progression, cellular plasticity, and stress response^14^. As part of the activated LINE-1 target-primed reverse transcription (TPRT) retrotransposition mechanism, the endonuclease activity of the LINE-1 ORF2 protein generates a double-strand DNA break that recruits repair proteins to the retrotransposon insertion site. Thus, DNA damage caused by overexpression of ORF2 proteins may induce genotoxic stress and apoptosis^14^. It has been proposed that LINE-1 hypomethylation and activation may play a causative role in urothelial carcinogenesis by inducing genetic instability which accompanies cancer progression, especially in high-stage and high-grade cancer^15^. However, non-invasive and early invasive papillary transitional cell carcinomas of stages pTa and pT1, respectively, already present a plethora of genetic aberrations including losses and gains of their genetic material^16,17^, with an increase of genomic instability when tumors progress further^17^.

Despite clear evidence for the involvement of an abnormal methylome and particularly LINE-1 hypomethylation in urothelial carcinogenesis it remains unknown how this process is initiated. To date, suitable i*n vitro* models to investigate this issue are lacking, but are urgently required to understand both, how these epigenetic alterations are caused and how they promote cancer progression.

Recently, we have provided evidence that aberrant epigenetic regulation of key enzymes of methyl group and polyamine metabolism could be involved in establishing aberrant methylomes in UC^18^. Here we show for the first time that experimental downregulation of the gene encoding a key enzyme of polyamine biosynthesis, ornithine decarboxylase (ODC1), results in global LINE-1 hypomethylation, induction of LINE-1 transcripts, double-strand DNA breaks in primary cultured uroepithelial cells and the immortalized uroepithelial cell line HBLAK. Similarly, urothelial carcinoma cells undergo apoptosis after having acquired double-strand DNA breaks following *ODC1* interference by siRNA.

## Results

### *ODC1* RNA interference rapidly induces LINE-1 hypomethylation and LINE-1 transcripts in primary cultures of uroepithelial cells

Recently, during a genome-wide screening of pTa and pT1 urothelial cancer tissue samples for altered DNA methylation, we observed distinct hypermethylation at the promoters of key genes of methyl group and polyamine metabolism pathways^18^. Disturbances of these key enzymes are known to lead to grave imbalances in the delicate intracellular SAM:SAH ratio resulting in genome wide DNA methylation alterations, including genome-wide LINE-1 hypomethylation^19–21^. Therefore, we hypothesized that our observation may provide an explanation for the mechanisms involved in hypomethylation of LINE-1 retrotransposons, a hallmark of early urothelial cancer.

**Figure 1:**
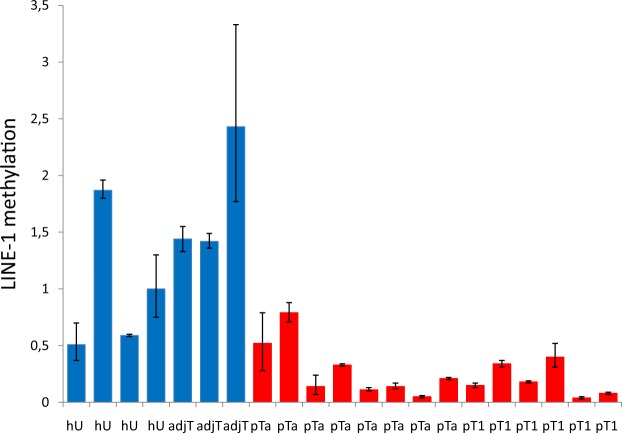
Relative quantification of LINE-1 methylation in early UC tissue specimens. LINE-1 methylation was measured by real time IDLN-MSPCR in four healthy-(hU), in three tumor adjacent uroepithelial (adjT), in eight pTa and in six pT1 UC tissue specimens.

First, we analyzed the LINE-1 methylation status of 8 pTa and 6 pT1 early urothelial cancer tissue specimens in which we had previously observed *ODC1* promoter hypermethylation^18^, using idiolocal normalized real-time Methylation Specific PCR (IDLN-MSP)^22^. This improved method allows for a reliable comparison of LINE-1 methylation in normal and tumor tissue specimens, despite genetic heterogeneity and copy number alterations present in early urothelial cancer^16,17^. We observed LINE-1 hypomethylation in 6 pTa and 6 pT1 urothelial carcinoma samples compared to 3 samples of healthy urothelium and 4 samples of tumor-adjacent uroepithelial tissue. Two pTa low grade tumor samples showed no hypomethylation (Fig. 1).

**Figure 2:**
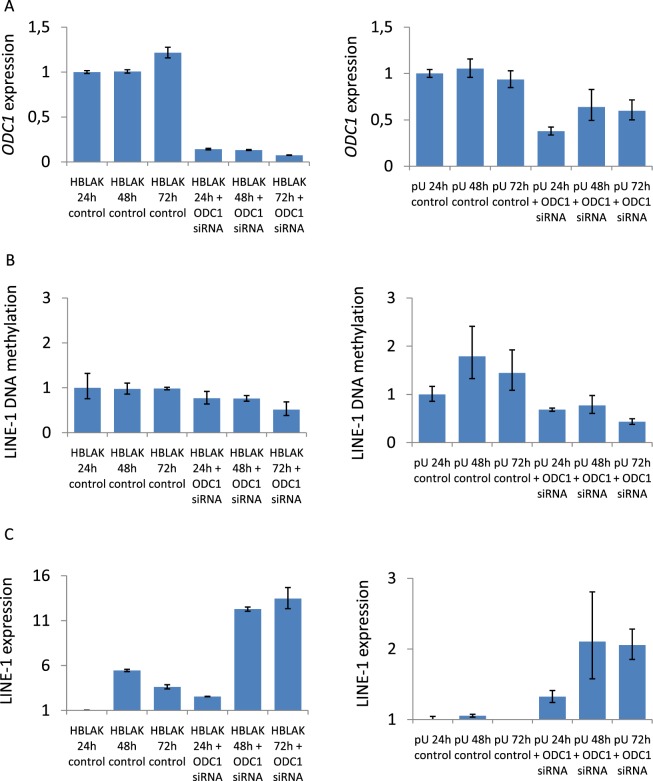
*ODC1* RNA interference in uroepithelial cells. *ODC1* gene expression (**A**), LINE-1 methylation (**B**), LINE-1 expression (**C**) in HBLAK, an immortalized uroepithelial cell line (graphs on the left) and in short-term cultured primary uroepithelial cells (graphs on the right) after downregulation of *ODC1* by RNAi over the indicated times.

**Figure 3:**
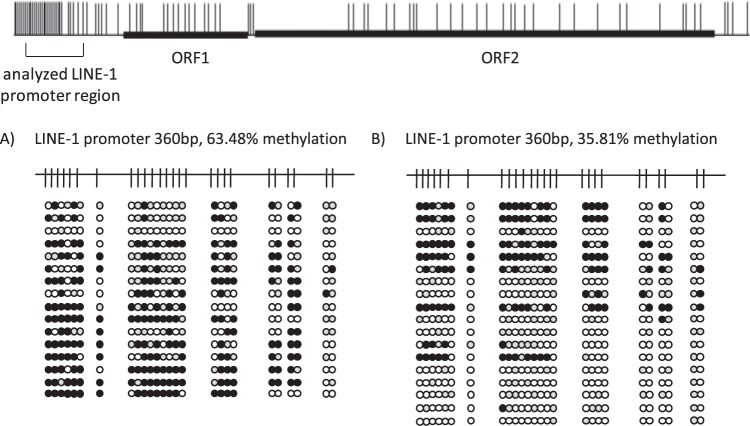
Bisulfite genomic sequencing of the LINE-1 promoter region in primary uroepithelial cells before and after *ODC1* RNA interference. Detailed analyses of LINE-1 promoter CpG methylation status of primary uroepithelial cells (**A**) and of the same uroepithelial cells after 72 h of treatment with *ODC1* RNAi (**B**). Black, white and grey circles stand for methylated, unmethylated and undefined CpG dinucleotides, respectiely. The graph at the top of the figure depicts a schematic representation of a full-length LINE-1 retroelement with the proportional distribution of all CpG dinucleotides (short ertical strokes) and the open reading frames 1 and 2 (ORF1/2). The CpG rich region analyzed by bisulfite genomic sequencing is highlighted by a square bracket.

Next, we down-regulated *ODC1* gene expression by RNAi in the immortalized uroepithelial cell line HBLAK. This cell line developed spontaneously from a primary culture of uroepithelial cells and has a stable karyotype with few chromosomal changes^23^. In addition, we applied this approach to primary, short-term cultured uroepithelial cell cultures, in order to exclude genetic and epigenetic alterations accumulating during prolonged cell cultivation as confounding factors. In both cell models, we achieved a clear repression of *ODC1* mRNA after 24 h of *ODC1* targeting by RNAi to <20% in HBLAK and <40% in primary uroepithelial cells (Fig. 2, suppl. Figure 1). ODC1 is tightly regulated at the protein level by multiple mechanisms which control its very rapid turnover^24^, implying that its transcriptional downregulation would lead to diminished enzyme activity within the cell. In addition, in both systems we detected a decrease in LINE-1 methylation, of 20% in HBLAK and of up to 50% in primary uroepithelial cell cultures after the first 24 h of *ODC1* downregulation. Consequently, LINE-1 transcripts increased after 48 h, with a 4-fold increase in HBLAK cells and a 2-fold increase in primary uroepithelial cell cultures. The increase in LINE-1 transcripts was paralleled by a decrease in DNA methylation of the LINE-1 promoter as demonstrated by bisulfite genomic sequencing of DNA from the same primary uroepithelial cell culture. DNA methylation in the *ODC1* RNAi-treated cells was 35.8% compared to 63.5% in non-targeting RNAi-treated cells (Fig. 3). Thus, repression of *ODC1* by RNAi rapidly results in LINE-1 hypomethylation and an increase of LINE-1 transcripts.

### *ODC1* RNA interference inhibits cell growth and induces p21 transcripts in uroepithelial cells as well as in UC cells

Deregulated expression of retrotransposons can damage the genome. In particular, the endonuclease activity of the LINE-1 ORF2 protein is able to generate double-strand DNA breaks, which can ultimately lead to apoptosis^14^, thus decreasing the number of viable cells.

**Figure 4:**
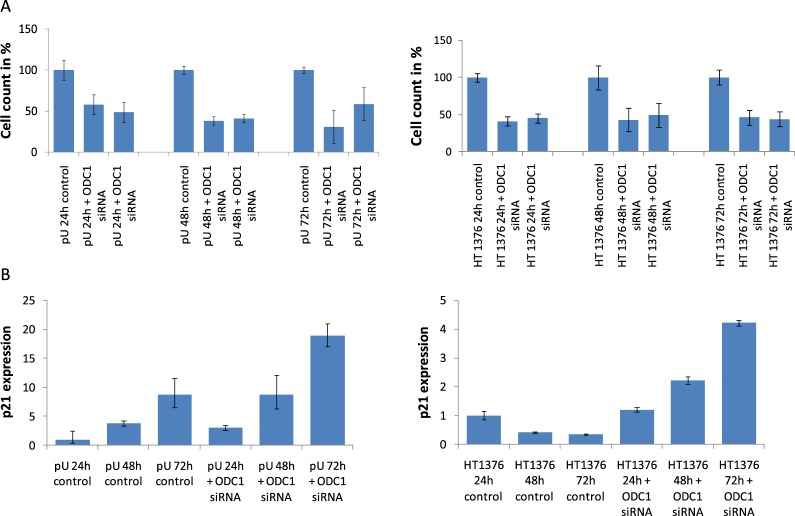
Cell growth and relative p21 expression in ODC1 RNAi treated uroepithelial cells and urothelial cancer cells. Cell numbers (**A**) and relative p21 expression (**B**) were measured after treatment of cultured uroepithelial cells (graphs on the left) and HT1376 UC cells (graphs on the right) by *ODC1* RNAi for 24, 48 and 72 h, as depicted.

Therefore we investigated cell numbers after *ODC1* RNA interference. We observed that after 24 h of RNAi treatment, uroepithelial cells undergo a substantial inhibition of cell growth (Fig. 4A). Interestingly, the same effect was observed as well in urothelial carcinoma cells (HT1376) (Fig. 4A). Expression of the cell cycle inhibitor p21 (WAF1/CIP1; *CDKN1A*) is induced by DNA damage^25^ leading to its binding and inhibition of cyclin dependent kinases CDK1 and CDK2 and inhibition of cell cycle progression^26^. This delay in the cell cycle enables the repair of damaged DNA. Concomitantly p21 exerts anti-apoptotic activities^25^, prominently by binding to and inhibiting caspase 3, a crucial executor of genotoxic stress-induced apoptosis^27^. Therefore, p21-deficient cells are more prone to undergo apoptosis after DNA damage^25^. Interestingly, data from TCGA suggest that p21 is significantly frequently mutated in bladder cancer^25^, a. o., the HT1376 UC cell line used here does not express p21 protein due to a frame shift mutation^28^.

Knockdown of *ODC1* by RNA interference diminished the number of cells in a uroepithelial cell culture by approximately 50% after 24 h. This effect persisted after 48 and 72 h of RNAi treatment (Fig. 4A). The decrease in cell numbers was accompanied by a 2-fold increase of p21 transcripts at all time points in siRNA-treated cells compared to control siRNA-treated cells, in which p21 mRNA notably increased over time (Fig. 4B).

In HT1376 UC cells a 4-fold increase occurs after 48 h of treatment that extended to an 8-fold increase after 72 h (Fig. 4B).

### *ODC1* RNA interference results in DNA double-strand breaks in uroepithelial cells and urothelial cancer cells and in apoptosis in urothelial cancer cells

**Figure 5:**
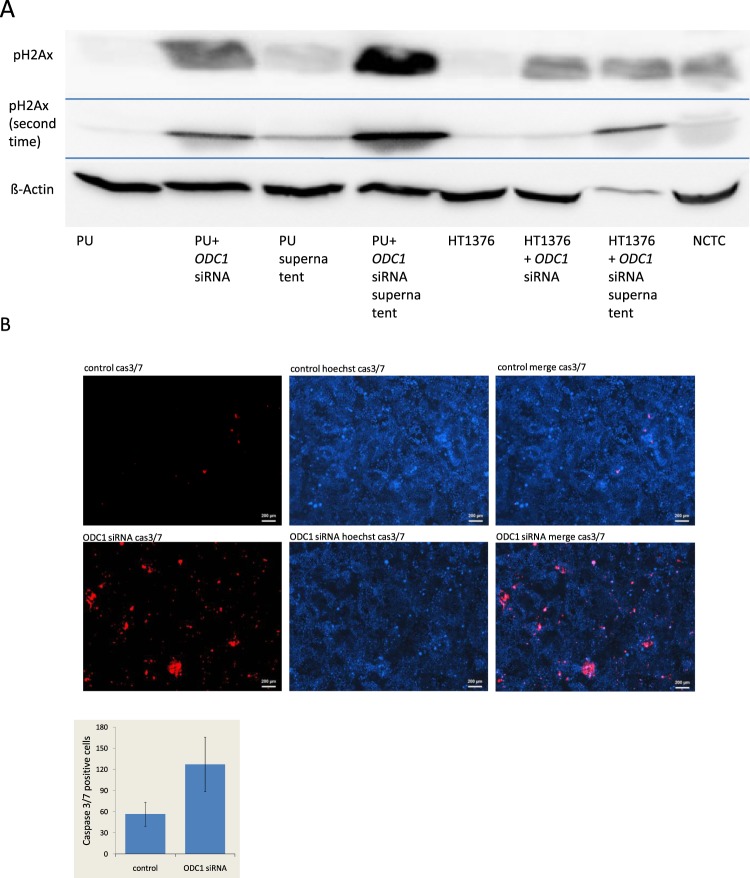
Detection of γH2Ax in uroepithelial and urothelial cancer cells (UC) and Caspase 3/7 actiity in UC after 72 h of *ODC1* downregulation by RNAi. γH2Ax was determined by Western Blot analysis in uroepithelial cells, urothelial cancer cells and their cell-free culture medium supernatants before and after treatment with *ODC1* RNAi for 72 h (**A**) (two analyses shown). Caspase 3/7 actiity was determined in HT1376 after the same treatment (**B**).

To investigate whether DNA damage and apoptosis may underlie the observed decrease in cellular viability following ODC1 downregulation, we assayed γH2AX, an established biomarker of DNA damage response at DNA double-strand breaks^29^. Consistently, we detected an increase of γH2AX in *ODC1* RNAi-treated uroepithelial cells after 72 h. Similar changes were observed in HT1376 urothelial cancer cells. Higher γH2AX levels were also detected in the supernatant of siRNA-treated cells compared to the controls (Fig. 5A). This is explained by loss of cell membrane integrity in apoptosis resulting in the release of the cellular contents, including chromatin into the environment^30,31^.

Finally, we investigated whether cell death indeed occurred by apoptosis by assaying the active form of caspase 3/7^30^. After 72 h of ODC1 RNAi treatment we detected more activated caspase 3/7 activity in the treated cancer cells than in the untreated HT1376 cells (Fig. 5B).

## Discussion

There is strong evidence that chemical carcinogens are involved in bladder carcinogenesis and induce mutations. However, it remains unclear which genes are affected first and constitute the crucial hits initiating the various subtypes of the disease. It is moreover unknown whether epigenetic alterations precede, accompany or follow genetic changes. Elucidating the first steps in bladder carcinogenesis could facilitate prevention and early diagnosis, and thereby lead to an improved prognosis and lower mortality rates. Previously, we showed that *ODC1* gene repression by promoter DNA methylation may be one of the first epigenetic hits in urothelial carcinogenesis (PrimeEpiHit (PEH) hypothesis)^18^.

ODC1 is the rate-limiting enzyme of polyamine biosynthesis and is expressed in virtually all tissues. It catalyzes the decarboxylation of ornithine to putrescine which subsequently is converted to the polyamines spermidine and spermine, by addition of propylamine groups from decarboxylated S-adenosylmethionine (dcSAM). Spermidine and spermin bind to and stabilize nucleic acids and nucleoprotein structures in the cell. In particular, they stabilize DNA and exert protection against cellular stress, for example, induced by DNA damaging agents^32^. Increased polyamine biosynthesis in cancer is considered important for tumor growth and survival due to increased metabolic needs^33^ and constitutes a critical factor in determining sensitivity to folate depletion^34^. Activation of ODC1 and consequently increased concentrations of polyamines are related to tumor promotion and progression in many cancer entities. For instance ODC1 overexpression has been reported for breast cancer, lung cancer, colon cancer, prostate cancer, pancreatic cancer, gliomas and others^33^. Difluoromethylornithine (DFMO), a potent irreversible inhibitor of ODC, exerts cytostatic activity by diminishing levels of folate-dependent metabolites, including S-adenosylmethionine (SAM) and thymidine pools in intestinal tumors and human colorectal cancer cells^35^ (compare suppl. Figure 2). Antitumor activity of DFMO has been supported by clinical trials on gliomas, nonmelanoma skin cancer, colon cancer and neuroblastoma^33^. Noteworthy, normal intestinal epithelial cells arrest in response to DFMO at the G1 phase of the cell cycle likely due to upregulation of p21^36^. Likewise, human melanoma cells develop a senescence-like phenotype following DFMO treatment^37^.

ODC1 is upregulated in many cancer types, as evidenced e.g. in the comprehensive pan-cancer “The Cancer Genome Atlas” TCGA expression data (suppl. Figure 3). However, ODC1 mRNA expression in bladder cancer is lower than in normal bladder tissues and among the lowest of any cancer type^38^ (suppl. Figure 3). In accord with these findings, in an investigation of patients with various genitourinary cancers, spermidine (20%) and spermine (20%) were diminished especially in the urine of bladder patients compared to probands without bladder cancer^39^.

DNA methylation is established as a mechanism regulating *ODC1* gene expression. In early studies a positive correlation between *ODC1* gene hypomethylation and expression was reported^40^ and experimental methylation of the *ODC1* gene abolished its expression^41^. Furthermore aberrant *ODC1* methylation has been reported in malignant cells^42^. Recently, we demonstrated that the 5′-regulatory region of *ODC1* is hypermethylated in most analyzed early UC (pTa/pT1) specimens and that its promoter activity can be efficiently repressed by DNA methylation^18^. Thus we concluded that dense DNA methylation, epigenetically impairs ODC1 expression. ODC1 is tightly linked to methyl group metabolism since interference with its enzymatic activity results in accumulation of SAH and decarboxylated S-adenosylmethionine (dcSAM), potent competitive inhibitors of methylation reactions that cause genome-wide DNA demethylation, as shown e.g. in human oral cancer cells^20^. Thus, hypermethylation of *ODC1* impairing *ODC1* gene expression in early urothelial cancer could act in a feed-forward fashion to promote further changes in methylation patterns, global DNA and LINE-1 hypomethylation. LINE-1 hypomethylation and activation which are established as widespread and early events in urothelial carcinogenesis would be facilitated by *ODC1* repression. LINE-1 expression in turn is thought to contribute to genetic instability during tumor progression. Mimicking *ODC1* gene repression in early stage UC and in uroepithelial cells by RNA interference *in vitro* indeed induced a comparable process as it elicited LINE-1 demethylation, LINE-1 transcriptional activation and DNA double-strand breaks. This resulted in increased p21 expression and proliferation inhibition indicating the activation of cellular checkpoints. Consequently, diminished proliferation was observed in normal uroepithelial cells, whereas UC cells underwent apoptosis following interference with ODC1. This suggests that normal urothelial cells can respond to imbalances in methyl-group and polyamine metabolism as well as DNA damage by appropriate mechanisms, e.g. checkpoint activation, which UC cells lack. Nevertheless, if ODC1 deficiency is a frequent phenomenon in urothelial carcinogenesis, later on in the course of tumor progression, tumor cells need to adapt to it, counter or overcome it by still unknown mechanisms, which may include the impediment of checkpoints characteristic of advanced bladder cancers. Obviously, this difference in response to ODC1 repression or inhibition may provide a therapeutic window, which deserves further exploration.

Interference with ODC1, a key gene of polyamine metabolism, results in global demethylation and genetic instability. Further, we see that this may be responsible for the induction of the carcinogenesis process. With this model in hand, future research will be able to address whether this apparently crucial interference with polyamine group metabolism contributes to tumorigenesis. In addition, we will perform SAM/SAH ratio quantification of urinary S-adenosylmethionine and S-adenosylhomocysteine by stable-isotope-dilution liquid chromatography-mass spectrometry^43^ from UC patients and healthy probands to estimate its value for early detection of UC. In addition, using the same model we will explore the role of other methyl group metabolism genes that we found concomitantly hypermethylated with *ODC1* in urothelial carcinoma, like *AHCY* and *AHCYL2*^18^. We therefore hypothesize that further genes of methyl group metabolism in addition to ODC1 contribute to disturbances of the methylome and thereby promote tumor progression. We are very eager to see whether the genetic integrity of these key genes mirrors DNA adduction caused by chemical carcinogens. The disruption of the genetic integrity of a gene can lead to promoter methylation and constitutive epigenetic gene silencing.

One additional aim is to develop a reliable non-invasive urinary UC diagnosis based on a combination of methylation biomarkers, e.g. LINE-1 hypomethylation and/or hypermethylated key genes, e.g. *ODC1*, *AHCY*, providing an early non-invasive detection method for the disease with high sensitivity and specificity.

Finally, LINE-1 hypomethylation and activation is a common phenomenon in many different cancers^12,44^. To our knowledge until now there was no approach available to deliberately induce this important epigenetic alteration in normal epithelial cells. Models analogous to ours may therefore yield insights in the relationship between epigenetic alterations and carcinogenesis in other cancer types as well. Our results highlight the question to which extent, presumably temporary, disturbances in methyl-group metabolism and polyamine biosynthesis contribute to LINE-1 hypomethylation in other cancers^12,44^. If so, the *ODC1* RNAi approach we describe here may be suitable to study the functional consequences of LINE-1 hypomethylation and its role in the carcinogenesis process of many other cancer entities as well.

## Methods

### Tissue samples

#### Cell lines and cell cultures

Cultivation of primary uroepithelial cells and HBLAK cells was performed as described by Swiatkowski *et al*. and Hoffmann *et al*.^45,46^. The UC cell line HT1376 was isolated from a high grade urothelial cancer of a 58 years old female patient^47^ and was kindly provided by the DSMZ (Braunschweig, Germany).

#### Preparation of DNA from formalin-fixed, paraffin-embedded (*FFPE*) tissue samples

All methods were carried out in accordance with relevant guidelines and regulations. We confirm that the experimental protocols were approved and informed consent was obtained from all participants. An appropriate ethics vote was granted by the Kantonale Ethikkommission Zürich on 22.02.2013, Ref. Nr. KEK-ZH-Nr 2012–0352. All samples were prepared as previously described in detail^18^.

The tissue samples were pathologically reviewed, microdissected and prepared as previously described^18^. In Fig. 1, from left to right, all pTa samples but sample number 4 (high grade) are low grade and all pT1 samples but sample number 1 (low grade) are high grade UC samples.

Relative Quantification of LINE-1 methylation in UC and reference samples by real-time MSP. Real time Methylation-Specific PCR of differentially methylated LINE-1 promoter regions was performed as follows: Bisulfite-converted DNA of the tissue samples served as template for amplification of methylated LINE-1 sequences in a normalized real time MSP approach for genetic imbalanced DNA specimens as described^22^. The primers used are listed in Table 1. The amplification conditions were denaturation at 95 °C for 10 min, followed by 40 cycles of 95 °C for 30 s, Tm for 40 s and 72 °C for 15 s.

**Table 1:** Primers for IDLN-MSP.

Primer Name	Sequence	Product Length (bp)	Tm (°C)
sL1met	5′-GCGCGAGTCGAAGTAGGGC-3′	193	61
asL1met	5′-CTCCGACCAAATATAAAATATAATCTCG-3′		
sL1control	5′-AGGTTTTATTTTTGGGGGTAGGGTATA-3′	207	58
asL1control	5′-CCCCTACTAAAAAATACCTCCCAATTAAAC-3′		

### ODC1 RNA interference

400.000 (primary urothelium) or 50.000 (HT1376) cells per well were seeded in 6-well plates (Sarstedt, Nümbrecht, Germany). 40 pmol of siRNA (2 µl) (Human ODC1 (4953) siRNA, M-006668-00-0005 - SMARTpool or siGENOME Non-Targeting siRNA Pool, D-001206-14-05, Dharmacon) were mixed with 248 μl Opti-MEM (1×) medium (Gibco). 5 μl of Lipofectamine 2000 (Dharmacon) were mixed with 245 μl Opti-MEM (1×) medium (Gibco). All mixes were incubated for 5 mins at RT. Then every siRNA mix was mixed together with the Lipofectamine mix and incubated for additional 20 mins at RT. Afterwards, 500 μl of the final mix was added to each well. Cells were kept in the incubator at 37 °C for 24 h, 95% humidity and 5% CO_2_. Retransfection with siRNA was performed every 24 h for three times.

### Bisulfite genomic sequencing

Bisulfite sequencing was performed following bisulfite conversion with the EpiTec Kit (Qiagen, Hilden, Germany) as described^48–50^. PCR primers for specific amplification of LINE-1 promoter sequences are listed in Table 2. The amplification conditions were denaturation at 95 °C for 13 min, followed by 35 cycles of 95 *°*C for 50 s, 51 *°*C for 45 s, and 72 *°*C for 30 s. We used 10–20 ng of bisulfite converted DNA, x µl of a 200 *μ*M dNTP Mix solution (Promega), 10 pm of each primer and 1.5 units of HotStarTaq DNA Polymerase (Qiagen) per reaction. The amplification product was 436 bp. The TA Cloning Kit (Invitrogen) was used for cloning of the amplification products according to the manufacturer’s instructions. Sequence evaluation was performed with the BigDye Terminator Cycle Sequencing Kit (Applied Biosystems) on a DNA analyzer 3700 (Applied Biosystems) using the M13-as primer. On average 30 clones were sequenced to obtain the methylation profile of one sample. All sequences were aligned using CLUSTLW from the Kyoto University Bioinformatics Center on http://www.genome.jp/tools/clustalw/ and all methylated CpGs were manually counted for every single CpG position.

**Table 2:** Primers for LINE-1 bisulfite genomic sequencing.

Primer Name	Sequence	Product Length	TM
s1LINEkonv1Ampl	5′-GGTTTATTTTATTAGGGAGTGTTAG-3′	436	51
as1LINEkonv1Ampl	5′-ACAAAAACAAACAAACCTCC-3′		

### RNA preparation, cDNA synthesis, and real-time PCR

RNA was prepared using the RNeasy Mini Kit (Qiagen) according to the manufacturer’s instructions. First-strand cDNA synthesis was performed from 1.5 μg RNA by reverse transcription using oligo(dT) (Promega) and Moloney murine leukemia virus reverse transcriptase (Promega) in a volume of 50 μL at 42 °C for 1 h. Real-time PCR was carried out with SYBR Green PCR Mastermix (Applied Biosystems) using 25 ng template cDNA. All reactions were run in triplicates on a StepOnePlus System (Applied Biosystems, Foster City, CA). Standard curves were generated using StepOne software v2.1 (Applied Biosystems). Relative changes in gene expression were calculated following the ΔΔCt-method with glyceraldehyde-3-phosphate dehydrogenase (GAPDH) mRNA as a standard. Primers were designed after excluding pseudogenes or other closely related genomic sequences which could interfere with specific amplification by amplicon and primer sequences comparison in BLAT sequence data base (https://genome.ucsc.edu/FAQ/FAQblat.html) Table 3.

**Table 3:** Primers for real-time PCR.

Primer Name	Sequence	Product Length (bp)	TM (°C)
s1GAPDHmrna	5′-CATGACAACTTTGGTATCGTGGA-3′	381	62
as1GAPDHmrna	5′-GTGGGTGTCGCTGTTGAAGTC-3′		
s1ODC1mrna	5′-CCGCTCGAGCGGATAAGTAGGGAGCGGCGTG-3′	276	54
as1ODC1mrna	5′-ATCATGGCGACCCTACTCTTAC-3′		
s1LINEmRNA	5′-TGGAATAGGTGTGGTGTGGTGCT-3′	246	61
as1LINEmRNA	5′-TCACTCAGGACAGCCCAGACG-3'		
sp21mrna	5′-GGAAGACCATGTGGACCTGT-3′	146	55
asp21mrna	5′-GGCGTTTGGAGTGGTAGAAA-3′		

### Determination of cell numbers

After siRNA treatment the cell numbers were determined using a Neubauer counting chamber (Bürker, Friedrichsdorf, Germany). 10 μl of each cell suspension was mixed with 10 μl trypan blue (0.4%) (Gibco) and pipetted into the chamber. Four large corner squares were counted under an inverse laboratory microscope (Leica). Cell numbers were calculated with the following formula: cell/ml = average of the number of cells counted over all squares × chamber factor × dilution factor.

### Western blot

Primary uroepithelial cells and HT1376 urothelial cancer cells and NCCIT teratocarcinoma cells were lysed in lysis buffer containing 5 M NaCl, 1% NP-40, 0.5% DOC, 0.1% SDS, 1 mM EDTA, 50 mM Tris, pH 8.0, and freshly added 10 μL/mL protease inhibitor (Sigma, Munich, Germany). 15 μg of protein was resolved in a 12% sodium dodecyl sulfate-PAGE gel and transferred onto Immobilon-P membrane (Millipore). Membranes were probed with primary antibody against Phospho-Histone H2A.X (Ser139) (20E3, Rabbit mAb, Cell Signaling, Frankfurt, Germany) and β-Actin (8H10D10 Mouse mAb, Cell Signaling, Frankfurt, Germany) at 4 °C overnight, washed with 0.1% Tween-20 in Tris-buffered saline, and incubated with secondary antibody conjugated with horseradish peroxidase. The signals were visualized with enhanced luminescence (WesternBright Quantum, Advansta, Ort, Land).

### Apoptosis detection

Apoptosis assay was performed using the ImageIT Live Red Caspase-3 and -7 Detection Kit (Thermo Fisher Scientific, Ort, Land) according to the manufacturer's instructions. In brief, cells were seeded in a 12 well plate (Sarstedt, Ort, Land). After treatment with siRNA for 72 h, the cells were washed with PBS and incubated with a 30-fold dilution of the provided FLICA reagent for 60 min at 37 °C for 24 h, 95% humidity and 5% CO_2_. After this incubation the cells were washed again with PBS and nuclei were stained with HOECHST 33342 (Thermo Fisher Scientific). To remove the Hoechst solution two additional washing steps with a 1x dilution of the provided wash buffer were carried out for 5 mins at RT and cells were counted using a fluorescent microscope X-Cite Series 120 (Lumen Dynamics, Ort, Land).

## Supplementary information

Supplementary information.
