# Supplementary material for: Basic Hallmarks of Urothelial Cancer Unleashed in Primary Uroepithelium by Interference with the Epigenetic Master Regulator ODC1
Source: Sci Rep. 2020 Mar 2;10:3808. doi: 10.1038/s41598-020-60796-8 (PMC7052216; doi:10.1038/s41598-020-60796-8)
Supplement: Supplementary file 1 — Supplementary information. [file 41598_2020_60796_MOESM1_ESM.pdf]

Basic Hallmarks of Urothelial Cancer Unleashed in Primary Uroepithelium by Interference with the Epigenetic Master Regulator *ODC1*

Lars Erichsen[1], Hans-Helge Seifert[2], Wolfgang A. Schulz[3], Michèle J. Hoffmann[3], Günter Niegisch[3], Marcos J. Araúzo-Bravo[4,5], Marcelo L. Bendhack[6], Cedric Poyet[7], Thomas Hermanns[7], Agnes Beermann[1], Mohamed Hassan[8,9], Lisa Theis[1], Wardah Mahmood[1], Simeon Santourlidis[1]*

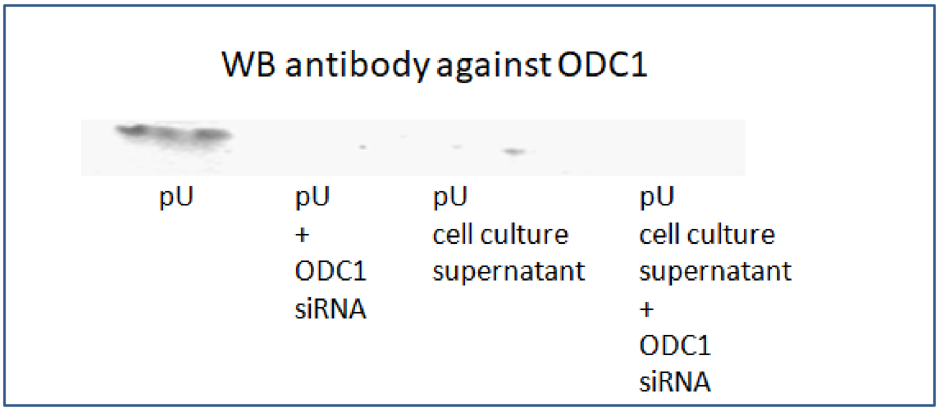

Suppl. Fig. 1: Western blot analysis in primary uroepithelial cell culture (pU) with antibody against ODC1 before and after 72h of treatment with ODC1 RNAi

A

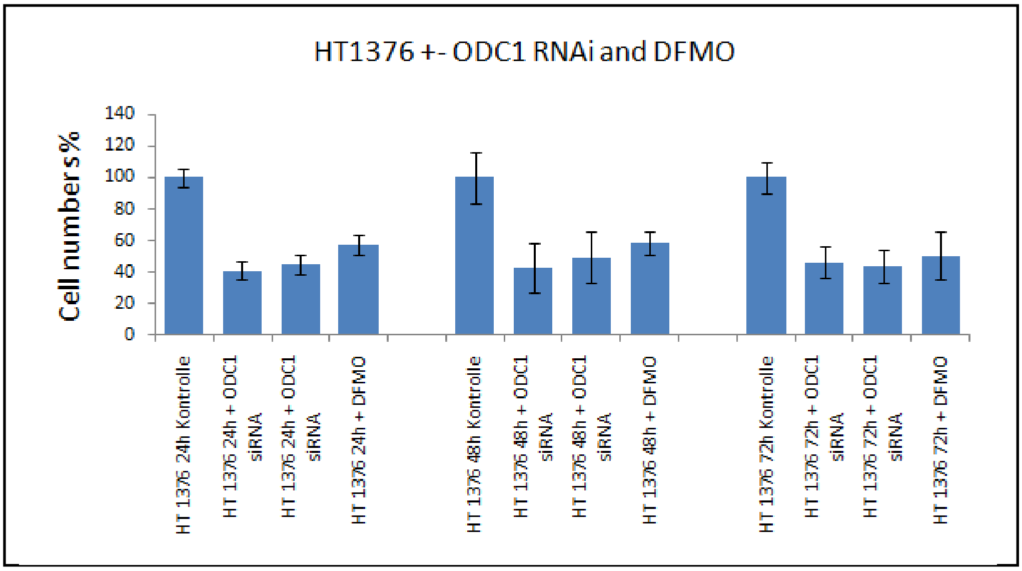

B

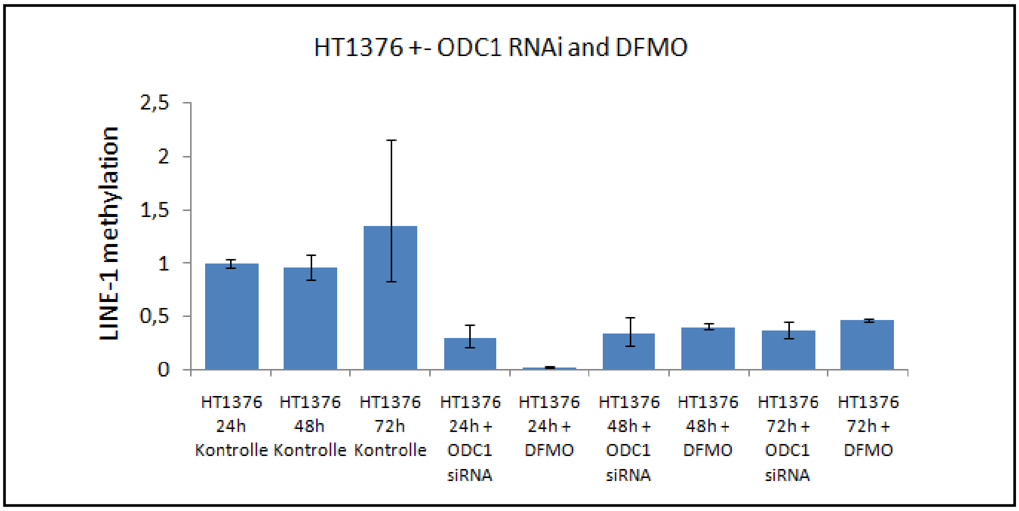

Suppl. Fig. 2: Effects of the specific ODC1 inhibitor DFMO on HT1376

HT1376 uroepithelial cancer cells were treated with DFMO (15µmol/l) before and after ODC1 RNAi treatment for the indicated time periods and cell viability (A) and LINE-1 methylation (B) were measured.

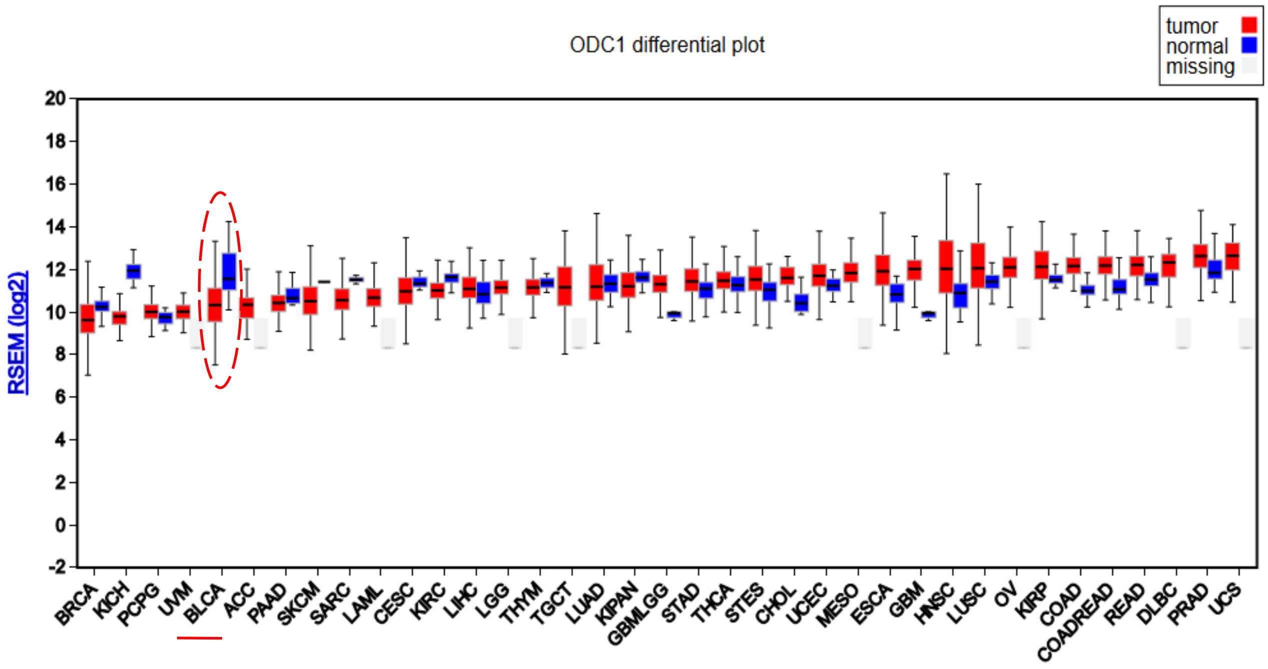

Suppl. Fig. 3: *ODC1* mRNA expression in diverse tumor entities.

The figure shows *ODC1* mRNA expression in diverse tumor tissues (red) in comparison to the respective healthy tissues (blue) quantified by RNA-seq (TCGA), analyzed by RNA sequencing Expectation-Maximization as described by Li and Dewey (2011) and plotted as $\log_2$ RSEM. The mean score for each cohort is indicated by a horizontal black bar. Missing data are indicated by white boxes. Tumor types are arranged from left to the right by increasing expression. For instance, bladder cancer samples show the fifth lowest *ODC1* expression and uterine carcinosarcoma shows the highest *ODC1* expression. For bladder cancer, *ODC1* expression in 408 cancer samples is compared to 19 samples of healthy urothelial tissue. They were 408 chemotherapy-naive, invasive, high-grade urothelial tumors (T1 [n = 1], T2–T4a, N0–3, M0–1) (Robertson et al., 2017). Note lower expression of *ODC1* in bladder tumors compared to healthy urothelial tissue (marked red).
